# Branch Migration Prevents DNA Loss during Double-Strand Break Repair

**DOI:** 10.1371/journal.pgen.1004485

**Published:** 2014-08-07

**Authors:** Julia S. P. Mawer, David R. F. Leach

**Affiliations:** Institute of Cell Biology, School of Biological Sciences, University of Edinburgh, Kings Buildings, Edinburgh, United Kingdom; Universidad de Sevilla, Spain

## Abstract

The repair of DNA double-strand breaks must be accurate to avoid genomic rearrangements that can lead to cell death and disease. This can be accomplished by promoting homologous recombination between correctly aligned sister chromosomes. Here, using a unique system for generating a site-specific DNA double-strand break in one copy of two replicating *Escherichia coli* sister chromosomes, we analyse the intermediates of sister-sister double-strand break repair. Using two-dimensional agarose gel electrophoresis, we show that when double-strand breaks are formed in the absence of RuvAB, 4-way DNA (Holliday) junctions are accumulated in a RecG-dependent manner, arguing against the long-standing view that the redundancy of RuvAB and RecG is in the resolution of Holliday junctions. Using pulsed-field gel electrophoresis, we explain the redundancy by showing that branch migration catalysed by RuvAB and RecG is required for stabilising the intermediates of repair as, when branch migration cannot take place, repair is aborted and DNA is lost at the break locus. We demonstrate that in the repair of correctly aligned sister chromosomes, an unstable early intermediate is stabilised by branch migration. This reliance on branch migration may have evolved to help promote recombination between correctly aligned sister chromosomes to prevent genomic rearrangements.

## Author Summary

Genetic recombination is critically important for the repair of DNA double-strand breaks and is the only repair mechanism available to the bacterium *Escherichia coli*. Repair requires that the appropriate location on an unbroken sister chromosome is recognised as a repair template, and this can be accomplished by a system that detects the presence of extensive DNA sequence identity. We show here that the two known branch migration activities of the cell, RuvAB and RecG, provide alternative mechanisms for stabilising early recombination intermediates. In their absence, broken DNA is extensively degraded at the site of the break consistent with abortion of recombination. It has previously been proposed that RuvABC and RecG can substitute for each other in the resolution of four-way Holliday junctions, whereas we show that they play a synergistic role in the formations of these junctions. Our results demonstrate that branch migration provides a mechanism capable of stabilising recombination intermediates when extensive DNA sequence homology is available, a reaction that may contribute to ensuring that repair occurs at an appropriate location on a sister chromosome.

## Introduction

Homologous recombination (HR) is a mechanism of DNA double-strand break repair (DSBR) that is conserved from bacteria to humans [1]. It involves resection of the broken DNA ends to generate single-stranded DNA overhangs, coated in a recombinase, which search the genome for homologous sequences and catalyse a reaction termed strand-invasion [2]. The product of strand-invasion is a joint molecule (JM), containing multiple DNA duplexes and frequently comprised of D-loops and Holliday junctions (HJs), also referred to as 3-way and 4-way DNA junctions, respectively. From the JM, DNA synthesis is established to restore the genetic information lost as a result of the break. Once synthesis is complete, the JM is resolved to generate the recombinant products of repair. When strand-invasion occurs between DNA sequences that are not fully homologous, such as between regions of repetitive DNA located on the same or different chromosomes, gross chromosomal rearrangements can occur. In higher organisms, where repetitive sequences are known to make up a substantial proportion of the genome, gross chromosomal rearrangements are associated with cancer [3], [4], [5]. This suggests that mechanisms exist for ensuring the correct pairing of sister chromosomes during HR.

**Figure 1:**
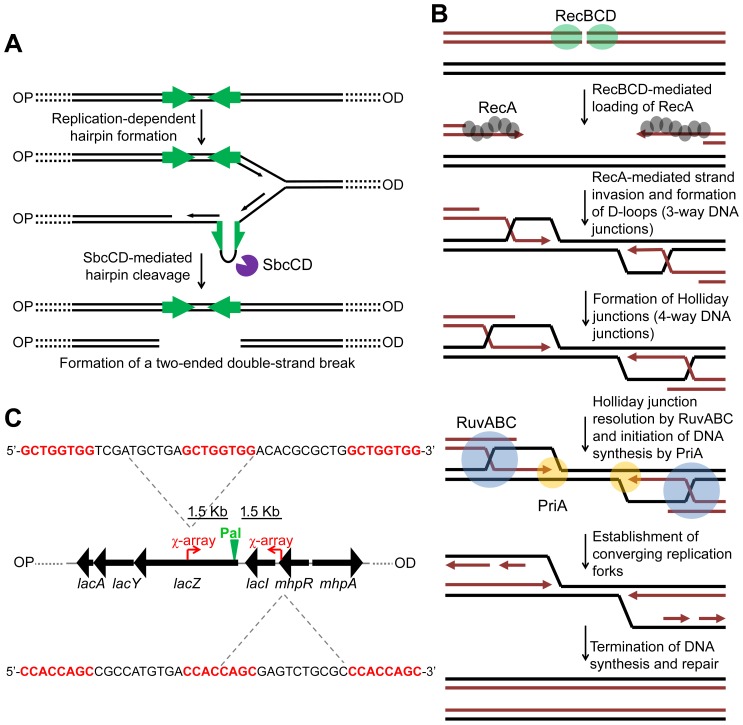
Making and repairing a site-specific DNA double-strand break in the *E. coli* chromosome. (A) SbcCD-mediated cleavage of a 246 bp interrupted palindrome inserted into the chromosomal *lacZ* gene. During replication, the palindrome becomes transiently single-stranded on the lagging-strand template. This allows it to form a DNA hairpin that is cleaved by SbcCD, generating a two-ended DSB. OP and OD indicate origin-proximal and origin-distal sides of the break, respectively. The palindrome is highlighted by green arrows. (B) RecBCD-mediated HR. The ends of the break are processed by RecBCD to generate 3′ ssDNA overhangs coated in RecA. RecA searches the genome for a homologous DNA sequence and catalyses strand-invasion. This forms a D-loop and HJs. The D-loop is acted upon by the replisome assembly factor, PriA, which initiates DNA synthesis. The HJs can be acted upon by RuvABC, branch-migrated and resolved. This generates two converging replication forks, which, upon convergence, terminate the repair process. (C) Map of the *lacZ* region of the *E. coli* chromosome illustrating the position and sequence of two 3x χ arrays that have been inserted 1.5 Kb either side of the palindrome in order to stimulate recombination in close proximity of the DSB. The 8 bp χ recognition sequence, highlighted in red, is repeated three times. OP and OD indicate origin-proximal and origin-distal sides of the break, respectively. Pal represents the position of the palindrome.

In order to gain further insight into the mechanism of HR, it is necessary to be able to detect different intermediates of repair as they are formed in live cells. To achieve this, it is desirable to work with a system for generating a site-specific DNA double-strand break (DSB) that can be efficiently repaired by HR with an unbroken sister chromosome. Such a system was described in 2008 in *Escherichia coli* [6]. This system uses an inducible hairpin endonuclease, SbcCD, to cleave a DNA hairpin that forms on the lagging-strand template following replication of a 246 bp interrupted palindrome that has been inserted into the chromosomal *lacZ* gene (Figure S1). Despite the fact that *E. coli* has a single origin of chromosomal DNA replication, this cleavage reaction generates a two-ended DSB at *lacZ* (Figure 1A) implying that cleavage occurs post-replication [6]. We distinguish the two sides of the break as origin-proximal (OP) and origin-distal (OD), also labelled OP and OD in all relevant figures (Figure S1). The DSB was shown to be efficiently repaired by RecBCD-mediated HR (Figure 1B) [6].

In order to accumulate intermediates of repair generated by this system, it is necessary to prevent their resolution. In *E. coli*, the proteins RuvABC and RecG have been implicated in resolving intermediates of HR. HJs are branch migrated by RuvAB and resolved via cleavage mediated by RuvC [7], [8], [9], [10]. Due to a strong synergistic effect of mutations in the *ruv* and *recG* genes in the efficiency of conjugational recombination, P1 transduction and survival following exposure to ionizing radiation and ISceI-mediated DSBs, a functional overlap of these proteins has been proposed, suggesting that RecG may also be implicated in resolving HJs [11], [12]. Throughout this paper we use the term resolution in its general sense of converting a molecule containing HJs to one without (i.e. resolution can be by branch migration, DNA replication, or cleavage). In support of a role of RecG in resolution, *in vitro* experiments have shown that both RuvABC and RecG process the same synthetic DNA junctions [13], [14]. Additionally, *in vivo* suppression of *ruv* mutations, by expression of the cryptic HJ resolvase RusA, also requires RecG [15]. Furthermore, the *Mycobacterium tuberculosis* RecG homologue, MtRecG, was shown to process similar branched DNA junctions *in vitro* [16].

However, it is important to note that many different roles for RecG have been proposed in the literature. Early work has shown that RecG antagonises RecA-mediated strand-exchange [17], [18]. This was puzzling given that RecG promotes recombination and led to the proposal that RecG might facilitate RecA-mediated strand-exchange from a 3′ invading substrate while antagonising strand-exchange from a 5′ invading substrate [17], [19]. In subsequent work, it has been argued that RecG catalyses replication fork reversal following UV irradiation [20] and prevents over-replication caused by replication fork collision, by converting 3′ to 5′ single-strand flaps [21], [22], [23], [24]. Whether or not these proposed activities relate to the synergy of *recG* and *ruv* mutations has not been clear, and the diverse consequences of a single *recG* mutation, as well as the ability of the purified protein to process many different substrates, have generated a complex picture of RecG's biological role.

Using the palindrome-based system for inducing DSBR between sister chromosomes, we analyse the intermediates of repair accumulated in the absence of the *ruv* and *recG* genes to elucidate their function during DSBR and gain further insight into the precise mechanism of repair. We show that RuvABC is the main HJ branch migration and resolution complex in *E. coli* and that RecG is required for the formation of HJs, by converting 3-way DNA junctions (D-loops) to 4-way DNA junctions (HJs). We go on to show that in the absence of both RuvAB and RecG, DNA is lost at the breakpoint due to an inability of a Δ*ruvAB* Δ*recG* mutant to catalyse branch migration. We conclude that branch migration, catalysed by either RuvAB or RecG, is essential for stabilising intermediates of DSBR by promoting the conversion of 3-way DNA junctions into 4-way DNA junctions, a conclusion that can explain the synergistic behaviour of *ruv* and *recG* mutants. We propose that this mechanism for stabilising intermediates favours DSBR reactions that occur between correctly aligned sister chromosomes, thus serving as a mechanism for ensuring correct pairing of sisters and, in turn, accurate repair of DSBs.

## Results

### RecG converts 3-way DNA junctions into 4-way DNA junctions

**Figure 2:**
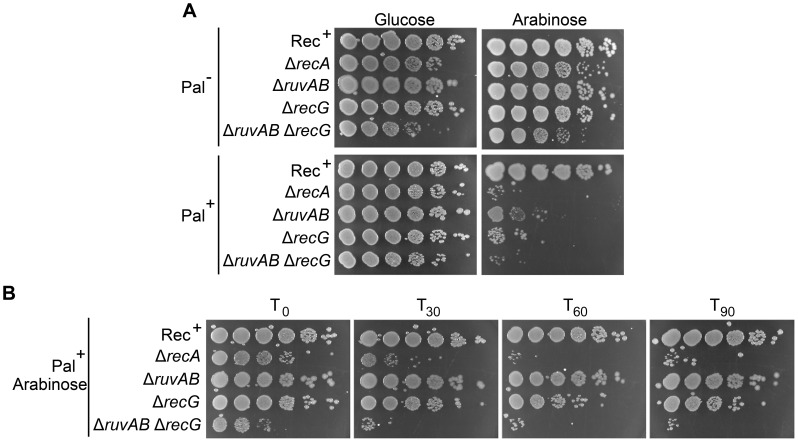
Viability of strains containing the palindrome, grown in 0.2% arabinose. (A) Chronic exposure to DSBs. Serial dilutions of strains were spotted on LB-agar plates supplemented with either 0.2% arabinose or 0.5% glucose and incubated overnight at 37°C. (B) Acute exposure to DSBs. Serial dilutions of strains containing the palindrome and grown in 0.2% arabinose for either 0, 30, 60, or 90 minutes were spotted on LB-agar plates supplemented with 0.5% glucose and incubated overnight at 37°C. Strains used; Rec^+^ Pal^+^ (DL2006), Rec^+^ Pal^−^ (DL2573), Δ*recA* Pal^+^ (DL2075), Δ*recA* Pal^−^ (DL2605), Δ*ruvAB* Pal^+^ (DL2801), Δ*ruvAB* Pal^−^ (DL2800), Δ*recG* Pal^+^ (DL2511), Δ*recG* Pal^−^ (DL2610), Δ*ruvAB* Δ*recG* Pal^+^ (DL4464), Δ*ruvAB* Δ*recG* Pal^−^ (DL4465).

*ruv* and *recG* mutants have been shown to be sensitive to DNA damage and this sensitivity is exacerbated in *ruv recG* double mutants [11], [12]. In accordance with these studies, DNA damage induced by SbcCD-mediated cleavage of a palindrome caused a loss of viability in single Δ*ruvAB* or Δ*recG* mutants that was severely exacerbated in the double Δ*ruvAB* Δ*recG* mutant (Figure 2 and Figure S2). Presumably, the decrease in viability is a consequence of the accumulation of toxic DNA repair intermediates that would normally be processed by these proteins.

**Figure 3:**
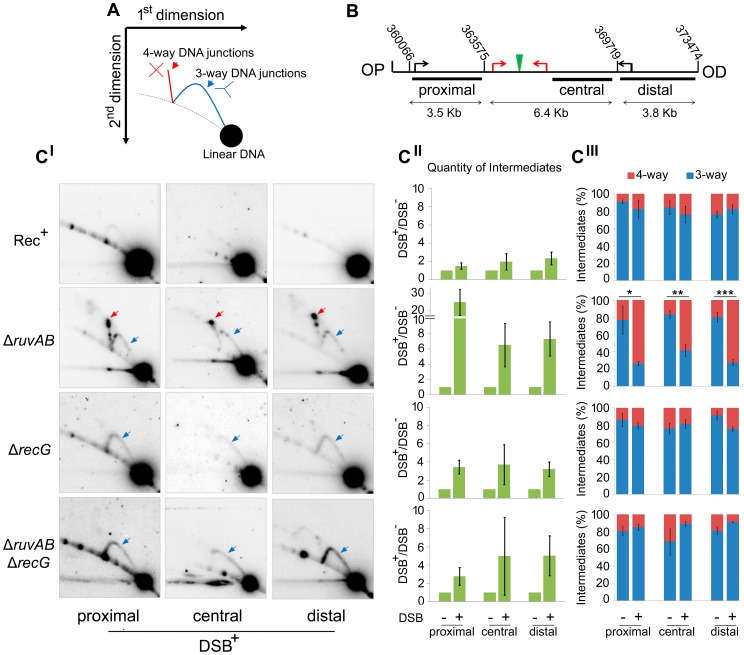
Intermediates of DSBR by 2D agarose gel electrophoresis. (A) Schematic representation of a 2D gel illustrating the expected positions of 3-way (blue) and 4-way (red) DNA junction migration. (B) Map of the region of the chromosome showing the relative positions of the palindrome (green), the 3x χ arrays (red arrows), endogenous χ sites (black arrows) and the chromosomal coordinates of the relevant MfeI and SacI restriction sites used to generate the proximal, central and distal fragments. The relative position of probes used is indicated by black rectangles. OP and OD indicate origin-proximal and origin-distal sides of the break, respectively. (C_I_) 2D gels of the proximal, central and distal fragments for Rec^+^ (DL4184), Δ*ruvAB* (DL4243), Δ*recG* (DL4311), and Δ*ruvAB* Δ*recG* (DL4260) strains containing the palindrome, exposed to 0.2% arabinose for 60 minutes. 3-way and 4-way DNA junctions are highlighted by a blue and red arrow, respectively. (C_II_) Quantifications (represented as mean ± SEM where n = 3) of total amount of intermediates (3-way plus 4-way DNA junctions) accumulated by 2D gel electrophoresis. (C_III_) Quantifications (represented as mean ± SEM where n = 3) of 3-way and 4-way DNA junctions. Statistical analysis was carried out using an unpaired T-test. * represents p<0.05, ** represents p<0.01 and *** represents p<0.005.

To detect these hypothetical repair intermediates and determine their structures, constructs containing three repeats of the crossover hotspot instigator, Chi (χ), were integrated 1.5 kb either side of the palindrome in order to enrich for recombination intermediates in close proximity of the DSB (Figure 1C). Subsequently, DNA from strains containing these constructs was isolated, digested with restriction endonucleases, and separated by two-dimensional (2D) agarose gel electrophoresis; a useful technique for distinguishing between 3-way DNA junctions and 4-way DNA junctions (Figure 3A). Three fragments surrounding the DSB were detected using radioactive probes (Figure 3B). All membranes were exposed for the same amount of time and intermediates were quantified relative to linear DNA (Figure 3C and S3). As shown in Figures 3C^II^, an increase in intermediates was detected in Δ*ruvAB*, Δ*recG* and Δ*ruvAB* Δ*recG* mutants specifically in conditions in which DSBs were induced (DSB^+^) relative to a very low background of spontaneous intermediates detected in the absence of induced breaks (DSB^−^) (Figure S3). A Δ*ruvAB* mutant, accumulated a significant amount of 4-way junctions, presumably HJs, when DSBs were induced (Figure 3C^I^; red arrows and 3C^III^). This was not the case when DSBs were induced in either Δ*recG* or Δ*ruvAB* Δ*recG* mutants (Figure 3C^I^ and 3C^III^). As 4-way junctions accumulated in a Δ*ruvAB* mutant but not in a Δ*ruvAB* Δ*recG* mutant, this suggests that RecG cannot simply be required for the resolution of 4-way junctions and must be required for their formation; presumably by catalysing the conversion of 3-way to 4-way junctions, an activity that has been reported for RecG *in vitro* [19], [20], [25], [26]. It is interesting to note that the analysis of the Δ*ruvAB* mutant reveals the existence of preferred configurations of branched DNA, which are seen as spots on the 2D gels (Figure 3C^I^). The placement of these spots is reproducible suggesting that they reflect DNA structures that accumulate in preference to others. Further work is required to determine what these structures are and how they are formed. Spots on the 4-way junction spike may reflect asymmetrically placed single HJs or double HJs and spots on the 3-way junction arcs may reflect positions of preferential single-strand invasion or pausing of DNA synthesis. However, these 3-way junction spots do not simply correlate with the expected positions of single-strand invasion predicted by the positions of Chi (χ) sites. As 3-way junctions are expected to form early in the reaction via strand invasion, as well as later during re-synthesis of the broken DNA, further work is required to understand their provenance.

### The action of either RuvAB or RecG prevents loss of DNA at the breakpoint

**Figure 4:**
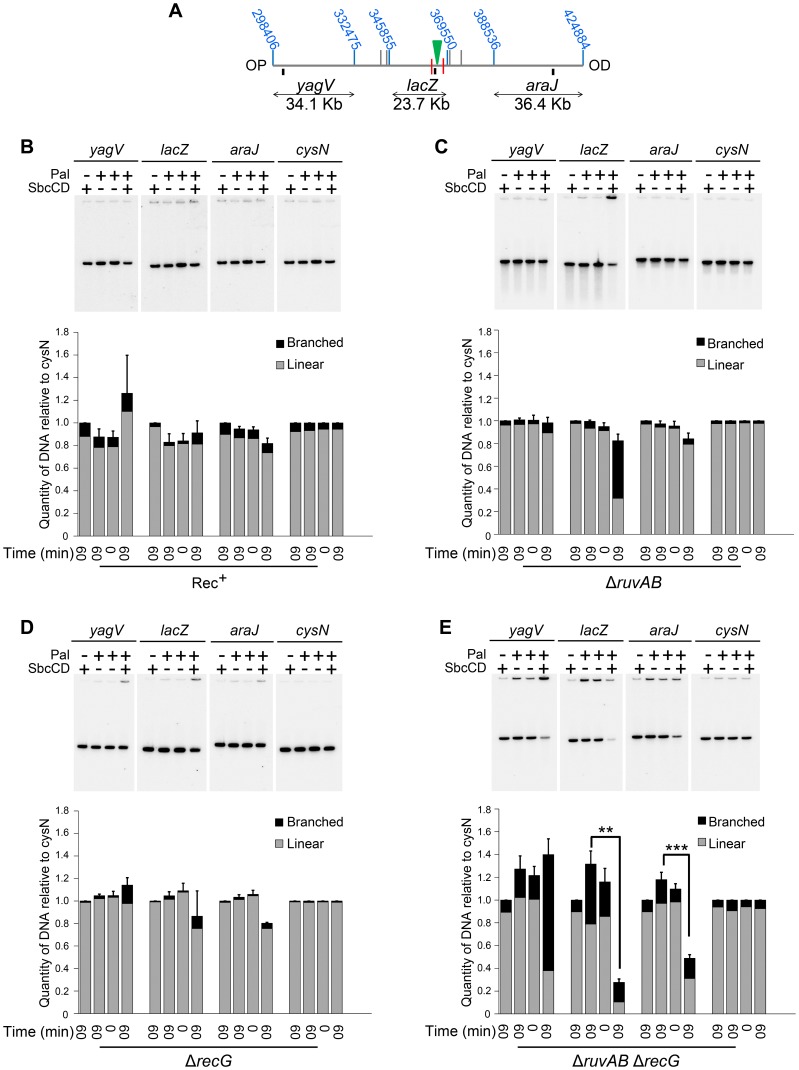
Intermediates of DSBR by PFG. (A) Map of the chromosome showing the three SalI fragments around the DSB. The coordinates of the restriction sites are shown in blue. The palindrome is shown as a green triangle and the 1.5 kb 3x χ arrays are shown as red lines. The relative position of probes are represented by small black rectangles. OP and OD indicate origin-proximal and origin-distal sides of the break, respectively. (B–E) PFGs for Rec^+^(DL4184 and DL4201), Δ*ruvAB* (DL4243 and DL4257), Δ*recG* (DL4311 and DL4312) and Δ*ruvAB* Δ*recG* (DL4260 and DL4313) strains, respectively. Quantifications are represented as mean ± SEM where n = 3. For each probe, Lane 1 contains DNA isolated from a strain not containing the palindrome, grown for 60 minutes in arabinose (pal^−^ SbcCD^+^ T_60_). Lane 2 contains DNA from a strain containing the palindrome, grown for 60 minutes in glucose (pal^+^ SbcCD^−^ T_60_). Lane 3 contains DNA from a strain containing the palindrome, prior to the addition of either glucose or arabinose (pal^+^ SbcCD^−^ T_0_). Lane 4 contains DNA from a strain containing the palindrome, grown for 60 minutes in arabinose (pal^+^ SbcCD^+^ T_60_). ‘Branched’ indicates signal from the well, ‘linear’ indicates signal from the gel. Quantifications are represented as mean ± SEM where n = 3. Statistical analysis was carried out using a paired T-test. * represents p<0.05, ** represents p<0.01 and *** represents p<0.005.

2D agarose gel electrophoresis is only suitable for analysing small chromosomal fragments (2–7 Kb). In order to determine whether intermediates of repair could be located across larger regions of the chromosome, pulsed-field gel electrophoresis (PFGE) was used as it allows the separation of big fragments of DNA. Additionally, branched DNA does not run into a pulsed-field gel (PFG), but remains trapped in the wells, and this allows it to be separated from its linear counterpart [27]. Plugs containing chromosomal DNA were digested to release three fragments surrounding the DSB (*yagV*, *lacZ*, and *araJ*) (Figure 4A). The total amount of DNA detected in these fragments (the sum of the signal from the gel and the well) was normalised to a control fragment, of a similar size, located on the opposite side of the chromosome (*cysN*) to account for differences in loading between samples. Additionally, the proportion of DNA that was retained in the wells of the gels was also measured as this DNA included the branched intermediates of repair (Figure 4B–E). In conditions of no DSBs (lanes 1, 2 and 3 for each probe), little DNA, of all the fragments probed, was retained in the wells (Figure 4B–E). A similar result was obtained when DSBs were induced in a recombination proficient strain (Figure 4B; lane 4 for each probe). Upon inducing DSBs in a Δ*ruvAB* mutant, a large proportion of the *lacZ* fragment, containing the DSB, was detected in the well of the gel whereas little of the *yagV* and *araJ* fragments appeared to contain branched DNA (Figure 4C). In a Δ*recG* mutant, DSB induction resulted in a small amount of branched DNA in all three fragments (Figure 4D). Unexpectedly, analysis of the DNA extracted from a Δ*ruvAB* Δ*recG* double mutant showed that when DSBs were induced, a significant amount of the DNA at the breakpoint (*lacZ* fragment) was lost (Figure 4E). It should be noted here that this result explained the low yield of DNA in the 2D gel analysis of the Δ*ruvAB* Δ*recG* double mutant. The reader should be aware that the DNA species obtained from the Δ*ruvAB* Δ*recG* mutant visualised using 2D gel electrophoresis (Figure 3), represent the minority of molecules recovered when DSBs were induced in that background.

The *lacZ* probe lies between the palindrome and the OP 1.5 Kb 3x χ array, in a region of DNA predicted to be degraded pre-RecBCD-mediated loading of RecA and strand-invasion. Therefore, loss of DNA in this region may suggest an inability of this mutant to initiate DNA synthesis associated with repair. However, a significant loss of DNA was also detected in the OD *araJ* fragment, which lies beyond the OD 1.5 Kb 3x χ array. This profile suggests that the loss of DNA observed may not be due to an inability to re-establish DNA synthesis, but due to an inability to form repair intermediates close to the DSB.

**Figure 5:**
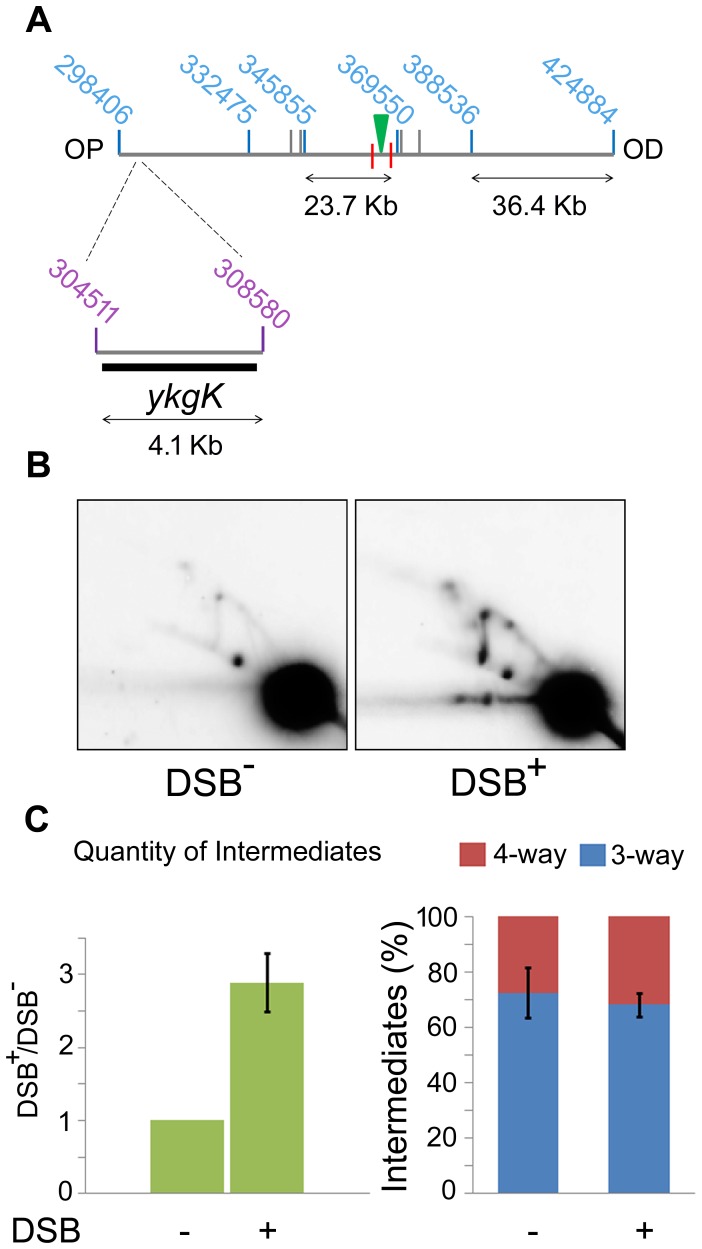
2D agarose gel electrophoresis 30 Kb upstream of the DSB. (A) SalI map of the region surrounding the DSB showing the location of the 4.1 kb *ykgK* fragment analysed by 2D agarose gel electrophoresis. Coordinates for the SalI restriction fragments detected in previous experiments are given in blue. Coordinates for the BspDI restriction fragment detected by 2D agarose gel electrophoresis are given in purple and the *ykgK* probe is shown as a black rectangle. The location of the palindrome is shown as a green triangle. The 1.5 kb 3x χ arrays are marked by red lines. OP and OD indicate origin-proximal and origin-distal sides of the break, respectively. (B) 2D agarose gel of Δ*ruvAB* Δ*recG* mutants containing (DSB^+^), or not (DSB^−^), the palindrome and grown in the presence of 0.2% arabinose for 60 minutes. Strains used were DL4260 (*lacZ*::*pal*) and DL4313 (*lacZ* ^+^). (C) Quantification (represented as mean ± SEM where n = 3) of intermediates accumulated in the strain containing the palindrome (DSB^+^), relative to the strain not containing the palindrome (DSB^−^) and the percentage of 4-way DNA junctions and 3-way DNA junctions accumulated in each strain.

Interestingly, in the OP *yagV* fragment, there was no loss of DNA but a dramatic accumulation of branched DNA. 2D agarose gel electrophoresis confirmed this accumulation of intermediates but revealed that there was still no bias towards the accumulation of either 3-way or 4-way DNA junctions when DSBs were induced, as was seen with the same mutant in the DNA remaining at the locus of the breakpoint (Figure 3C and Figure 5).

### Branch migration is required for preventing loss of DNA at the DSB

**Figure 6:**
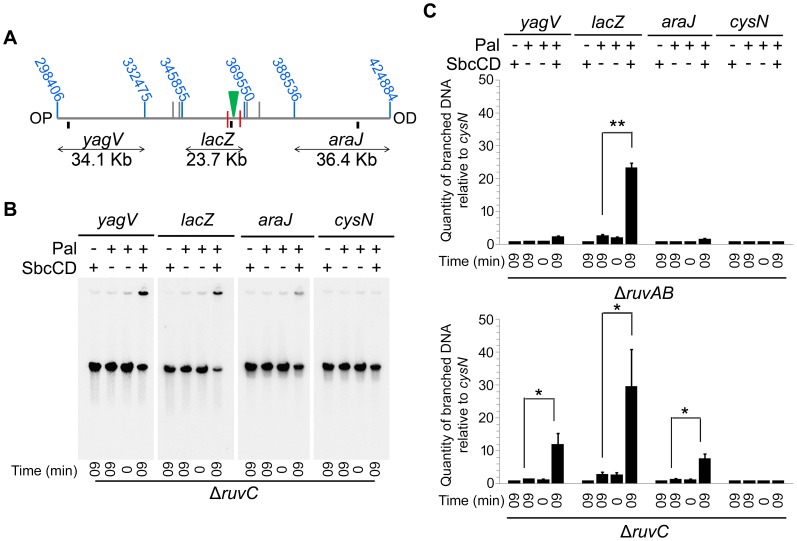
Detection of branch migration using PFGE. (A) Map of the chromosome showing the three SalI fragments around the DSB. The coordinates of the restriction sites are shown in blue. The palindrome is shown as a green triangle and the 1.5 kb 3x χ arrays are shown as red lines. The relative position of probes are represented by small black rectangles. OP and OD indicate origin-proximal and origin-distal sides of the break, respectively. (B) Gel of branched DNA retained in the wells of PFGs from DNA isolated from Δ*ruvC* (DL4913 and DL4914) mutants. Samples were run as in Figure 3. (C) Quantifications (represented as mean ± SEM where n = 3) of branched DNA retained in the wells of PFGs from DNA isolated from Δ*ruvAB* (DL4243 and DL4257) mutants (gel shown in Figure 3C) and Δ*ruvC* mutants (gel shown in panel B). Statistical analysis was carried out using a paired T-test. * represents p<0.05, ** represents p<0.01.

A Δ*ruvAB* Δ*recG* mutant, shown to lose DNA at the site of a DSB, is both unable to branch migrate and resolve HJs. In order to determine which of these activities is required to prevent the loss of DNA observed, a Δ*ruvAB* Δ*recG* mutant was compared to a Δ*ruvC* Δ*recG* mutant. A Δ*ruvC* Δ*recG* mutant still retains RuvAB and should therefore be able to catalyse branch migration. However, RuvAB cannot resolve HJs in the absence of RuvC, so HJs should remain unresolved in this background. The ability of RuvAB to catalyse branch migration in the absence of RuvC was confirmed by PFGE (Figure 6). A significant amount of branched DNA was accumulated in the wells of the PFGs in Δ*ruvAB* and Δ*ruvC* mutants (Figure 6C), consistent with the hypothesis that HJs are only resolved when all components of the RuvABC complex are present. However, the branched DNA accumulated in a Δ*ruvAB* mutant was located within the *lacZ* fragment containing the DSB, while in a Δ*ruvC* mutant, branched DNA was detected in all three fragments surrounding the break. This is indicative of RuvAB-mediated branch migration being active in the absence of RuvC.

**Figure 7:**
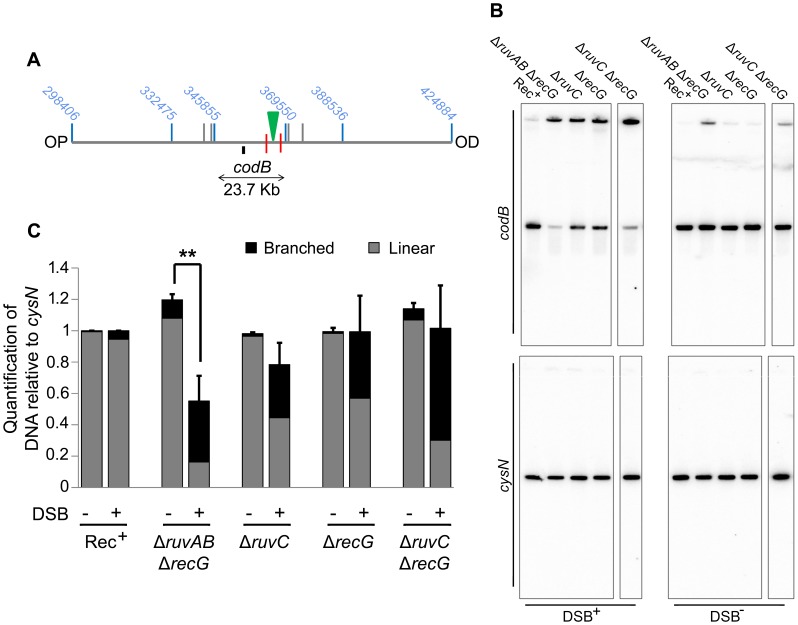
Detection of DNA loss in Δ*ruvAB* Δ*recG* and Δ*ruvC* Δ*recG* mutants. (A) Map of the chromosome showing the three SalI fragments surrounding the DSB. The coordinates of the restriction sites are shown in blue. The palindrome is shown as a green triangle and the 1.5 kb 3x χ arrays are shown as red lines. The relative position of the *codB* probe is represented by a small black rectangle. OP and OD indicate origin-proximal and origin-distal sides of the break, respectively. (B) Gels probed with *codB* probe and *cysN* probe. All strains were grown in the presence of 0.2% arabinose for 60 minutes. DSB^+^ strains contain the palindrome while DSB^−^ strains do not. Strains used were; Rec^+^ (DL4184 and DL4201), Δ*ruvAB* Δ*recG* (DL4260 and DL4313), Δ*ruvC* (DL4913 and DL4914), Δ*recG* (DL4311 and DL4312), Δ*ruvC* Δ*recG* (DL4941 and DL4942). All lanes shown for each probe were derived from the same membrane. (C) Quantification (represented as mean ± SEM where n = 3) of linear and branched DNA relative to Rec^+^. Statistical analysis was carried out using an unpaired T-test. ** represents p<0.01.

Once this was verified, PFGE was used to check whether a Δ*ruvC* Δ*recG* mutant lost DNA in response to DSBs and to compare this to DNA loss in a Δ*ruvAB* Δ*recG* strain (Figure 7). In order to detect DNA located OP of the DSB and beyond the point of initial RecBCD-mediated loading of RecA and strand-invasion, a new probe, *codB*, that binds 8.5 Kb OP to the 3x χ array, was designed (Figure 7A). Between the breakpoint and the *codB* probe, as well as the 1.5 Kb 3x χ array, there is an endogenous χ site located 5 Kb from the breakpoint, in the *cynX* gene. Assuming a 20%–35% probability of χ site recognition, these four χ sites should be responsible for between 59% and 82% of strand-invasion events [28], [29], [30]. As shown in Figure 7, DNA hybridising to the *codB* probe was lost in a Δ*ruvAB* Δ*recG* mutant when DSBs were induced, consistent with the hypothesis that intermediates of repair are not stable in this background. Interestingly, this loss did not occur in a Δ*ruvC* Δ*recG* mutant. These results imply that the loss of DNA observed in a Δ*ruvAB* Δ*recG* mutant is due to an inability to branch migrate intermediates of repair, rather than an inability to resolve HJs, and this results in the destabilisation of repair intermediates.

## Discussion

### RecG is required to convert 3-way to 4-way DNA junctions during DSB repair

Due to a synergistic effect of mutations in the *ruv* and *recG* genes, it had originally been argued that these proteins may provide alternative pathways for resolving HJs. We have corroborated the observation that mutations in both *ruvAB* and *recG* result in enhanced sensitivity to DSBs compared to the respective single mutations when DSBs are induced by SbcCD-mediated cleavage of a palindrome (Figure 2 and Figure S2). However, analysis by 2D agarose gel electrophoresis of the DNA at the DSB has confirmed that this enhanced sensitivity was not accompanied by an accumulation of HJs (4-way DNA junctions) (Figure 3C). This result argues against the view that RuvABC and RecG are simply redundant because they provide alternative pathways to resolve HJs. 4-way DNA junctions were indeed accumulated close to the DSB in a Δ*ruvAB* mutant, consistent with a role of RuvAB in processing HJs (Figure 3C). However, these 4-way junctions were not accumulated in proximity to the DSB in a Δ*ruvAB* Δ*recG* mutant, arguing that RecG is required for their formation.

### RuvAB and RecG provide alternative pathways for stabilising intermediates of repair

The use of PFGE for studying intermediates of DSBR revealed why 4-way DNA junctions were not accumulated close to the DSB in a Δ*ruvAB* Δ*recG* mutant. In the absence of both RuvAB and RecG, DNA was lost at the site of the DSB. This was accompanied by an accumulation of branched DNA over 30 Kb away from the breakpoint (Figure 4 and 5). For the DNA in the *lacZ* locus to be lost, and for intermediates of repair to be present in the *yagV* fragment, the OP DNA end must be processed, by RecBCD, from the *lacZ* fragment to the *yagV* fragment. This is surprising as RecBCD will encounter eight endogenous χ sites (as well as the OP 3x χ array) in the region of the chromosome between the DSB and the *yagV* fragment and should induce RecA-mediated strand-invasion as a result [31]. This suggests that in a Δ*ruvAB* Δ*recG* mutant background, the products of RecA-mediated strand-invasion are not stable, which allows RecBCD to process a region of the chromosome that would not be processed in a wild type context.

χ sequences around the *E. coli* chromosome are distributed asymmetrically to limit DNA end processing by RecBCD on the OP side of a DSB [32]. The asymmetry detected for OP accumulation of branched DNA and OD loss of DNA in a Δ*ruvAB* Δ*recG* mutant reflects this asymmetry of endogenous χ sequences, strengthening the hypothesis that the degradation is mediated by RecBCD. There are eight endogenous χ sites between the break and the OP *yagV* fragment that itself contains two χ sites and only one endogenous χ site between the break and the OD *araJ* fragment that contains no χ sites. We conclude that in a Δ*ruvAB* Δ*recG* mutant the products of strand-invasion are transient and non-productive for repair due to an inability to branch-migrate 3-way junctions and form 4-way junctions. This leads to the disruption of the 3-way junctions and the formation of a new DNA end for RecBCD to process. When the next χ site is recognised, a new event of strand-invasion is initiated, which is once again disrupted by a lack of branch migration activity. Over time, the broken chromosome is degraded.

We propose that in *ruvABC* ^+^ *recG* ^+^ cells, when sister chromosomes are correctly aligned, branch migration is facilitated and this stabilises intermediates of repair by promoting the formation of 4-way DNA junctions. This favours the accurate repair of DSBs. This interpretation is supported by the observation that the frequency of ectopic recombination is increased in *recG* mutant strains in a chromosomal direct repeat deletion assay [33], [34], [35] and in recombination between chromosomal and plasmid homologies [36]. In the direct repeat assay, this is the case unless the replicative helicase is compromised [33], [35].

### Implications for the proposed role of RecG in resolving HJs

The redundancy we observe in the stabilisation of JMs can explain the synergistic defect caused by *ruv* and *recG* mutations and this no longer necessitates the previously proposed redundancy in HJ resolution. However, redundancy at this stage cannot be excluded. Furthermore, if RuvABC and RecG do not provide alternative pathways for the resolution of HJs, such pathways must nevertheless exist otherwise *recG* and *ruv* mutations would be epistatic. This has led us to consider again the evidence that *recG* and *ruv* provide two pathways for HJ resolution. The strongest evidence in favour of this hypothesis is the observation that suppressors of the UV sensitivity of *ruv* mutations cause activation of the cryptic HJ resolvase, RusA, and this suppression requires RecG [15]. The simplest interpretation of this result is that the branch migration activity of RecG translocates HJs to positions where they are cleaved by RusA. However, RusA is not expressed in the absence of the activating mutation, *rus*, and no HJ resolvases other than RusA and RuvC have been discovered in *E. coli* [37]. Furthermore the requirement for *recG* in the suppression of *ruv* by *rus* can now simply be explained by the destabilisation of JMs that we observe in a *recG ruvAB* double mutant. If JMs are not formed, then they cannot be resolved by RusA.

This leaves the question of whether there exists a pathway to resolve HJs that is an alternative to cleavage by RuvABC. The genetics argue that this is so. *Ruv* mutants are only modestly recombination defective but *recG ruv* double mutants are as defective as *recA*. This is synergy, not epistasis, arguing that the presence of RuvABC or RecG can provide alternative ways of successfully catalysing recombination. If synergy is explained by redundancy of RuvAB and RecG at the stage of JM formation and RuvABC provides a way to resolve HJs then there must also be a way to resolve HJs in the absence of RuvABC. What is this route? The observation that HJ resolution in the absence of RuvABC leads to substantial yields of chromosome dimers [11], [27] demonstrates clearly that this pathway can generate crossover products and excludes models such as double HJ dissolution by branch migration that would produce only non-crossovers. It has been suggested that new rounds of DNA replication initiated at the chromosomal origin can sometimes pass through HJs and generate the resolved chromosomes [27]. To explain the synergy of *recG* and *ruv*, given the assumption that the activities were redundant for HJ resolution, it was suggested that RecG might facilitate this reaction. However, the results presented here open up the possibility that the replication forks that manage to pass through HJs may do so without the help of RecG.

It is clear from our work that HJs accumulate in a ruvAB mutant, implying that they persist long enough to be detected and the data shown in Figure 6 argue that JMs are not resolved before they can be branch migrated by RuvAB. These data are not well explained by an immediate role of RecG in HJ resolution but are compatible with a delay of resolution in the absence of RuvABC as predicted if resolution is mediated by the next round of DNA replication initiated at the chromosomal origin.

Many functions have been proposed for RecG, including the resolution of Holliday junctions [11], [12], replication fork reversal following UV irradiation [20], conversion of 3′ flaps to 5′ flaps in the termination of replication [21], [22], [23], [24], destabilisation of RecA promoted strand exchange [17], [18] and stabilisation RecA-promoted strand exchange [17], [19]. Our results clearly demonstrate the importance of the role of RecG, as an alternative to RuvAB, in stabilising RecA-promoted strand exchange in DSBR.

### Mechanisms for stabilising intermediates and promoting accurate repair of DSBs

**Figure 8:**
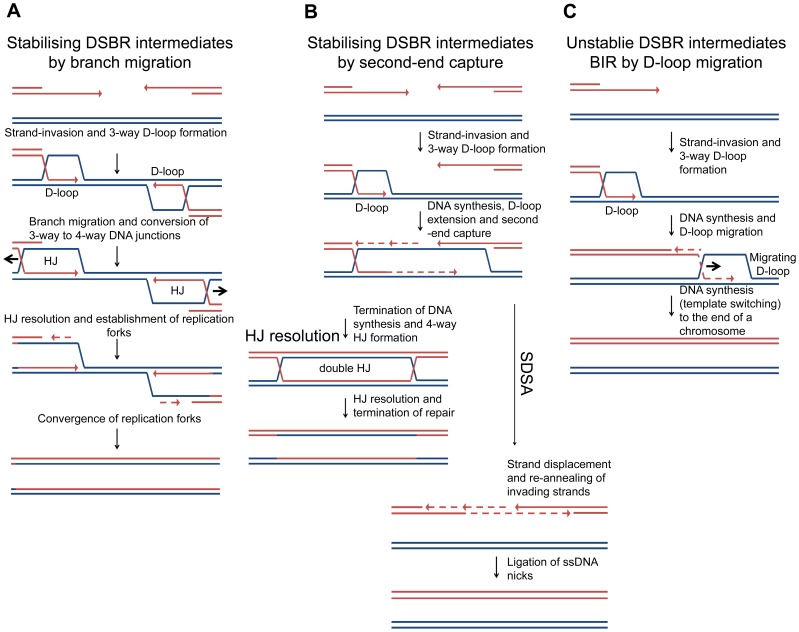
Models of DSBR. (A) Stabilising DSBR intermediates by branch migration. In *E. coli*, following extensive DNA degradation by RecBCD, a resected 3′ end invades a sister chromosome to establish a D-loop in a reaction catalysed by RecA protein. This is stabilised via branch migration catalysed by RuvAB or RecG to form a Holliday junction that can be resolved to generate a replication fork. Only one end is shown here, but a two-ended reaction can occur as shown in Figure 1. In the absence of branch migration (in a Δ*ruvAB* Δ*recG* mutant) the products of RecA-mediated strand-invasion (3-way D-loops) are unstable and non-proficient for repair. This results in extrusion of the invading end from the unbroken chromosome to re-generate a broken end. This end is processed by RecBCD and a second round of strand-invasion is initiated. The whole process is repeated. Over time the broken chromosome is degraded. (B) Stabilising DSBR intermediates by second-end capture. In the canonical eukaryotic DSBR pathway for the repair of a two-ended DSB, one of two 3′ ssDNA ends invades an intact DNA duplex, at a region of homology, to generate a 3-way DNA junction (D-loop). DNA synthesis is then primed off the 3′ DNA end and this leads to the extension of the D-loop, which eventually uncovers enough homology to allow second-end capture. This generates a stable dHJ intermediate, which is then resolved to generate the recombinant products of repair. Alternatively, the 3′ invading DNA strand is extended allowing second-end capture and then both invading strands are ejected and re-anneal in a reaction know as Synthesis Dependant Strand Annealing (SDSA). (C) Unstable DSBR intermediates for the repair of a one-ended DSB by BIR (by D-loop migration) in eukaryotic cells. The 3′ ssDNA ends invades an intact DNA duplex, at a region of homology, to generate a 3-way DNA junction (D-loop). DNA synthesis is primed off the 3′ end. As synthesis proceeds, the unstable D-loop migrates with the replication fork, resulting in the extrusion of the newly synthesised strand and conservative DNA replication. Template switching may occur. The reaction ends when the D-loop either reaches the end of a chromosomes or converges with an oncoming replication fork.

Many models for the repair of DNA DSBs have been proposed over the years and these are reviewed in detail by Pâques and Haber [38]. Some of the models predict the formation of 4-way DNA junctions, from 3-way DNA junctions, and some do not. Most models for the repair of two-ended DSBs in *Saccharomyces cerevisiae* implicate invasion of one DNA end followed by DNA synthesis that uncovers a region of homology to induce an event known as second-end capture. This can be processed to generate a double HJ intermediate that has been detected *in vivo* in meiotic and mitotic cells [39], [40], an intermediate that may be resolved by branch migration or HJ cleavage (Figure 8 – HJ resolution). Alternatively, the invading strands can be ejected and re-annealed, prior to the completion of the double-HJ structure, in a reaction known as synthesis-dependent stand-annealing (Figure 8B – SDSA), a mechanism that has the advantage of not generating crossover outcomes. If strand-invasion were to occur at short regions of homology, such as repetitive elements, rather than at correctly aligned sister chromatids or homologous chromosomes, second-end capture may be disfavoured. If it does occur, and resection proceeds beyond the region of homology, resolution by SDSA would minimise genome instability by ensuring non-crossover outcomes [41]. In *S. cerevisiae*, during the repair of a two-ended DSB in which second-end capture is prevented, the invading end can be repaired by break-induced replication (BIR) (see [42] for a recent review). BIR has been shown to involve multiple rounds of strand-invasion in the initial phase of the reaction, consistent with repair-intermediate instability [43]. Furthermore, BIR is mutagenic consistent with a D-loop migration mechanism in which short-lived mismatches are not corrected but, instead, are copied in a conservative mode of DNA replication [44], [45], [46] (Figure 8C). These observations suggest that second-end capture plays an important role in promoting accurate repair of two-ended DSBs. Indeed, second-end capture prevents BIR and promotes gene conversion through the operation of a recombination execution checkpoint (REC) that senses the proximity and orientation of the two recombining ends before DNA synthesis is initiated. When such ends are sensed, as is the case with a two-ended DSB, accurate repair is ensured and the outcome is directed towards gene conversion [47].

In contrast to DSBR in eukaryotes, in *E. coli*, DSBR involves extensive DNA degradation followed by the re-establishment of replication forks via the PriA-DnaB pathway of replisome loading [2], [48], [49]. This is understood to result in the formation of converging replication forks that restore the DNA between the two recombining ends (Figure 1B). Within this model of DSBR, the stabilisation of intermediates by second-end capture should not be possible. We suggest that branch migration is an alternative to second-end capture for stabilising an intermediate that can be then converted to a 4-way DNA junction.

The stabilisation of recombination intermediates by branch migration, which we have observed, is expected to work equally well for two-ended and one-ended DSBs. On the other hand, the stabilisation of intermediates determined in some way by second-end capture, by definition, cannot operate at one-ended DSBs. These types of DSBs do arise endogenously from replication forks that run into replication fork barriers, single-stranded DNA nicks or gaps, and from cleavage of reversed forks, and are thought to be the most common type of break encountered by all cells [50], [51], [52]. As second-end capture cannot be implicated as a mechanism for stabilising the intermediates generated from the repair of one-ended DSBs, this raises the intriguing question of how they can be stabilised in eukaryotic cells. The repair of one-ended sister chromatid breaks is distinguished from inter-chromatid BIR by the requirement of Rad51, Rad52, Rad54 and Rad59 [53] but little is known about the pathway of repair including how early intermediates are stabilised. One possibility is that some one-ended breaks await the formation of a second end produced by the firing of a replication origin situated on the other side of the causative lesion (i.e. a two-ended break is generated from the sum of two one-ended breaks occurring one on each side of the same inducing lesion (such as a persistent single-strand gap)). The mechanism discovered here presents a solution adopted by *E. coli* that is expected to work equally well at one-ended and two-ended breaks.

Repair of a DSB by HR with a sister chromosome has evolved to be accurate, despite the fact that genomes contain regions of repetitive sequence that could act as substrates for incorrect pairing. Here we show that the *E. coli* proteins RuvAB and RecG do not simply provide alternative pathways for the resolution of HJs, as previously suggested, but play redundant roles in stabilising recombination intermediates between sister chromosomes.

## Materials and Methods

### Strains

All strains used are listed in the supporting information. See Table S1 for a list of strains, Table S2 for plasmids used in the construction of the strains, Table S3 for oligonucleotides used in the construction of the plasmids and protocols S1 and S2 for methods used in the construction of the strains and plasmids.

### Induction of DSBs and isolation of chromosomal DNA in agarose plugs

Overnight cultures grown in 5 ml L-broth were diluted to an optical density (OD_600nm_) of 0.02 and grow at 37°C with agitation to an OD_600nm_ of 0.2. The P*_BAD_-sbcDC* construct was induced by adding 0.2% arabinose. If P*_BAD_-sbcDC* was to be repressed as well as induced, the culture (OD_600nm_ of 0.2) was split in two and either 0.5% glucose or 0.2% arabinose was added. Cultures were put back at 37°C to grow for 60 minutes. Cells were harvested at 4°C and washed 2X in TEN buffer (50 mM Tris, 50 mM EDTA, 100 mM NaCl, pH 8.0). Cells were re-suspended in TEN buffer to an OD_600nm_ of 80 (for 2D agarose gel electrophoresis) or an OD_600nm_ of 4 (for PFGE) and mixed with an equal volume of 0.8% (for 2D agarose gel electrophoresis) or 2% (for PFGE) low melting point agarose (Invitrogen) prepared in TEN buffer equilibrated to 50°C. The agarose/cell mix was poured into plug moulds (BioRad) and allowed to set. Plugs were treated in NDS solution (0.5 M EDTA, 10 mM Tris, 0.55 M NaOH, 36.8 mM lauroyl sarcosine; pH 8.0) supplemented with 1 mg/ml of proteinase K (Roche) and put at 37°C overnight. Fresh NDS + proteinase K was added for a second overnight and plugs were stored at 4°C in fresh NDS. To digest, a plug was washed in 1X restriction buffer for 6 hours, replacing the buffer every hour. The plug was placed in fresh 1X restriction buffer, supplemented with the restriction enzyme and incubated at 37°C overnight with rocking.

### 2D agarose gel electrophoresis

A plug digested with a restriction enzyme was run in the first dimension in 1X TBE (89 mM Tris-borate, 2 mM EDTA) on a 0.4% (w/v) agarose gel and run at 1 V/cm for 26 hours at 4°C. The lane was sliced out, rotated 90°, and set in the second dimension agarose (1% in 1X TBE supplemented with 0.3 µg/ml ethidium bromide). The second dimension was run at 6 V/cm for 10 hours at 4°C. The DNA was transferred to a positively charged nylon membrane by Southern blotting and cross-linked using UV-light.

### PFGE

A plug digested with a restriction enzyme was run on a 1% ultra high gel strength agarose (AquaPor) prepared in 0.5X TBE and run on a CHEF-DR II PFGE (BioRad) at 6 V/cm for 10 hours at 4°C. Switch time was set to 5–30 seconds with an inclusion angle of 120°. The DNA was transferred to a positively charged nylon membrane by Southern blotting and cross-linked using UV-light.

### Radioactive detection of DNA

DNA was detected using ^32^P α-dATP incorporated (using Stratagene Prime-It II random primer labelling kit) into a PCR fragment. Probes were hybridised to membranes overnight at 65°C in 10 ml of Church-Gilbert buffer (7% SDS, 0.5 M NaH_2_PO_4_, 1 mM EDTA, 1% BSA). Membranes were washed at 60°C in 2X SSC (1X SSC: 0.15 M NaCl, 0.015 M Na-citrate), supplemented with 0.1% SDS, for 15 minutes and then 0.5X SSC, supplemented with 0.1% SDS, for 30 minutes. Labelled membranes were exposed to GE healthcare storage phosphor screens and scanned using a Molecular Dynamics Storm 860 phosphor imager scanner. Images were quantified using GE healthcare ImageQuant TL. See Table S3 for the oligonucleotides used in the generation of the probes.

## Supporting Information

Figure S1 Map of the *E. coli* chromosome. The origin of replication (*oriC*) is marked in red while the terminus (*dif*) is marked in blue. The relative position of *lacZ* is marked by a black arrow and the palindrome is highlighted in green. The origin-proximal (OP) and origin-distal (OD) sides of the palindrome are labelled accordingly.
(TIF)

Figure S2 Effect of an SbcCD-mediated DSB on the growth rate of recombination deficient strains. Growth curves (represented as mean ± SEM where n = 3) of strains with or without the palindrome (Pal^+^ and Pal^−^, respectively), grown for 300 minutes in conditions that either induce the expression of *sbcDC* (arabinose) or repress it (glucose). Strains used; Rec^+^ Pal^+^ (DL2006), Rec^+^ Pal^−^ (DL2573), Δ*recA* Pal^+^ (DL2075), Δ*recA* Pal^−^ (DL2605), Δ*ruvAB* Pal^+^ (DL2801), Δ*ruvAB* Pal^−^ (DL2800), Δ*recG* Pal^+^ (DL2511), Δ*recG* Pal^−^ (DL2610), Δ*ruvAB* Δ*recG* Pal^+^ (DL4464), Δ*ruvAB* Δ*recG* Pal^−^ (DL4465).
(TIF)

Figure S3 2D agarose gel electrophoresis of DSB^−^ condition. (A) Map of the chromosome showing the three SalI fragments around the DSB. The coordinates of the restriction sites are shown in black. The palindrome is shown as a green triangle and the 1.5 kb 3x χ arrays are shown as red arrows. Endogenous χ sites are shown as black arrows. The relative position of probes are represented by black rectangles. OP and OD indicate origin-proximal and origin-distal sides of the break, respectively. (B) Control 2D gels of strains not containing the palindrome, grown in the presence of 0.2% arabinose for 60 minutes. (DSB^+^ blots are shown in Figure 3C). Strains used; Rec^+^ (DL4201), Δ*ruvAB* (DL4257), Δ*recG* (DL4312), and Δ*ruvAB* Δ*recG* (DL4313).
(TIF)

Table S1 Table of *E. coli* strains.
(DOCX)

Table S2 Table of plasmids.
(DOCX)

Table S3 Table of primers.
(DOCX)

Protocol S1 Construction of strains.
(DOCX)

Protocol S2 Construction of pDL4137 and pDL4138.
(DOCX)
