# Supplementary figures and images for: Branch Migration Prevents DNA Loss during Double-Strand Break Repair
Source: PLoS Genet. 2014 Aug 7;10(8):e1004485. doi: 10.1371/journal.pgen.1004485 (PMC4125073; doi:10.1371/journal.pgen.1004485)

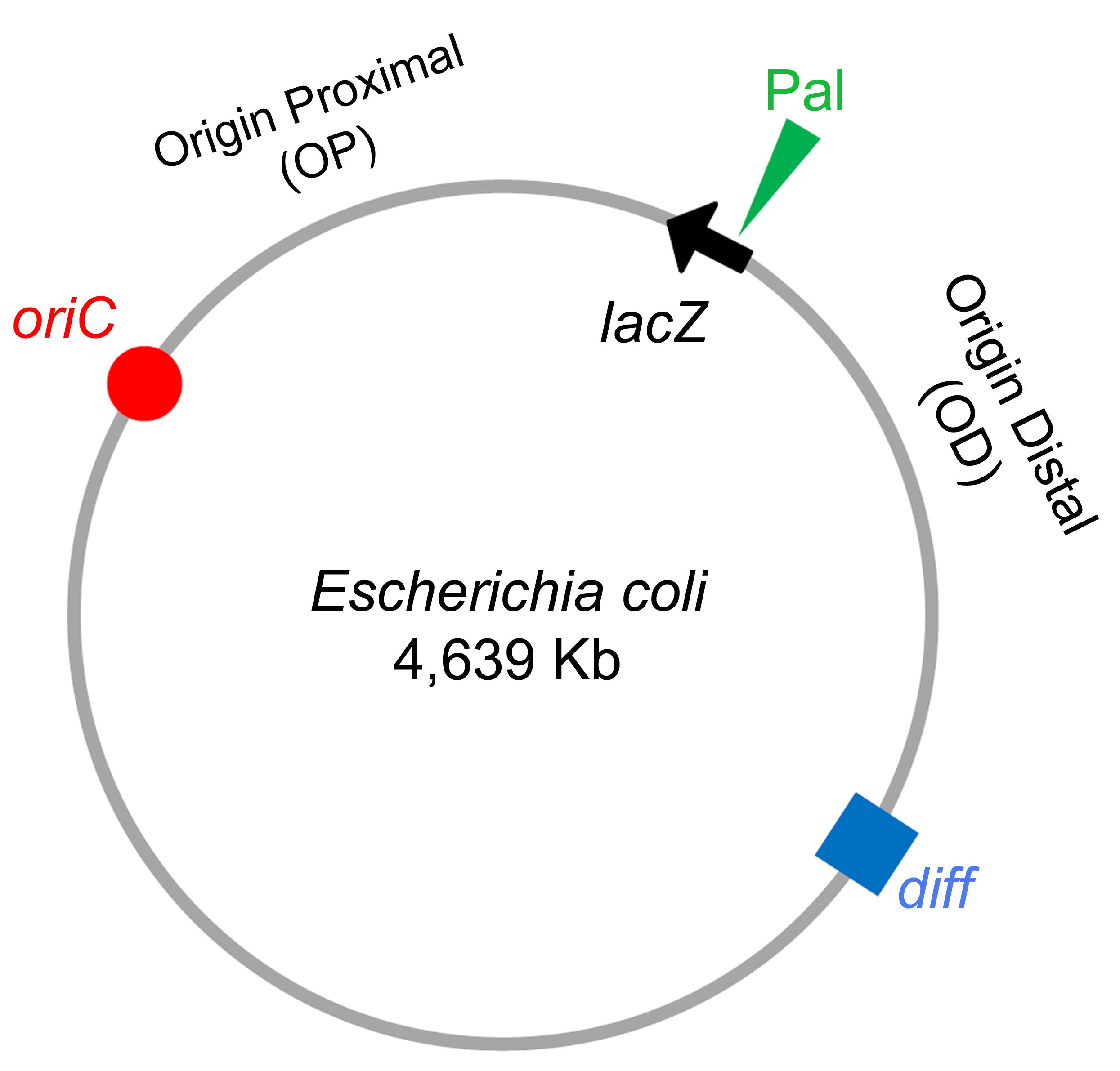

Supplement: Figure S1 — Map of the E. coli chromosome. The origin of replication (oriC) is marked in red while the terminus (dif) is marked in blue. The relative position of lacZ is marked by a black arrow and the palindrome is highlighted in green. The origin-proximal (OP) and origin-distal (OD) sides of the palindrome are labelled accordingly. (TIF) [file pgen.1004485.s001.tif]

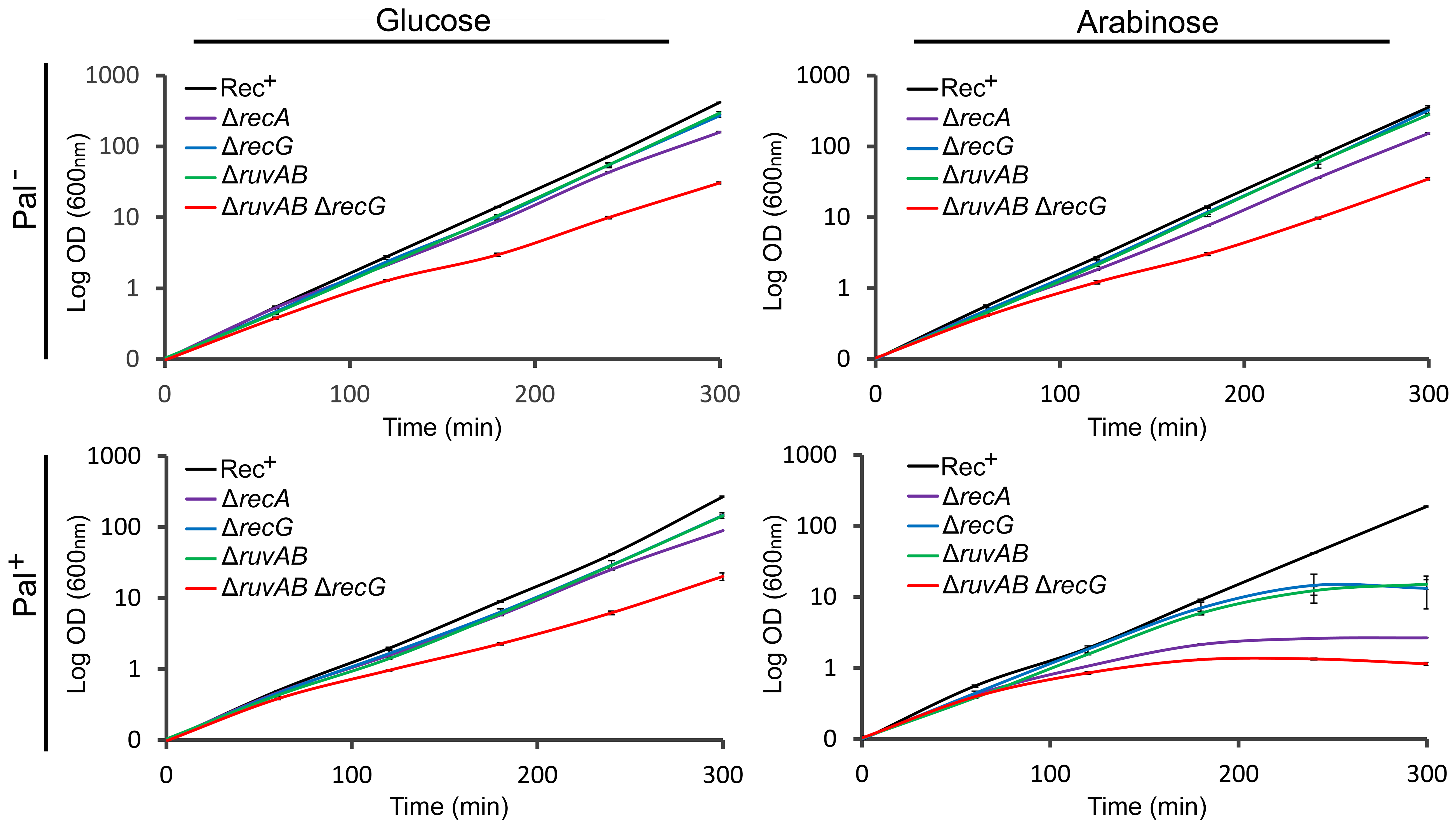

Supplement: Figure S2 — Effect of an SbcCD-mediated DSB on the growth rate of recombination deficient strains. Growth curves (represented as mean ± SEM where n = 3) of strains with or without the palindrome (Pal+ and Pal−, respectively), grown for 300 minutes in conditions that either induce the expression of sbcDC (arabinose) or repress it (glucose). Strains used; Rec+ Pal+ (DL2006), Rec+ Pal− (DL2573), ΔrecA Pal+ (DL2075), ΔrecA Pal− (DL2605), ΔruvAB Pal+ (DL2801), ΔruvAB Pal− (DL2800), ΔrecG Pal+ (DL2511), ΔrecG Pal− (DL2610), ΔruvAB ΔrecG Pal+ (DL4464), ΔruvAB ΔrecG Pal− (DL4465). (TIF) [file pgen.1004485.s002.tif]

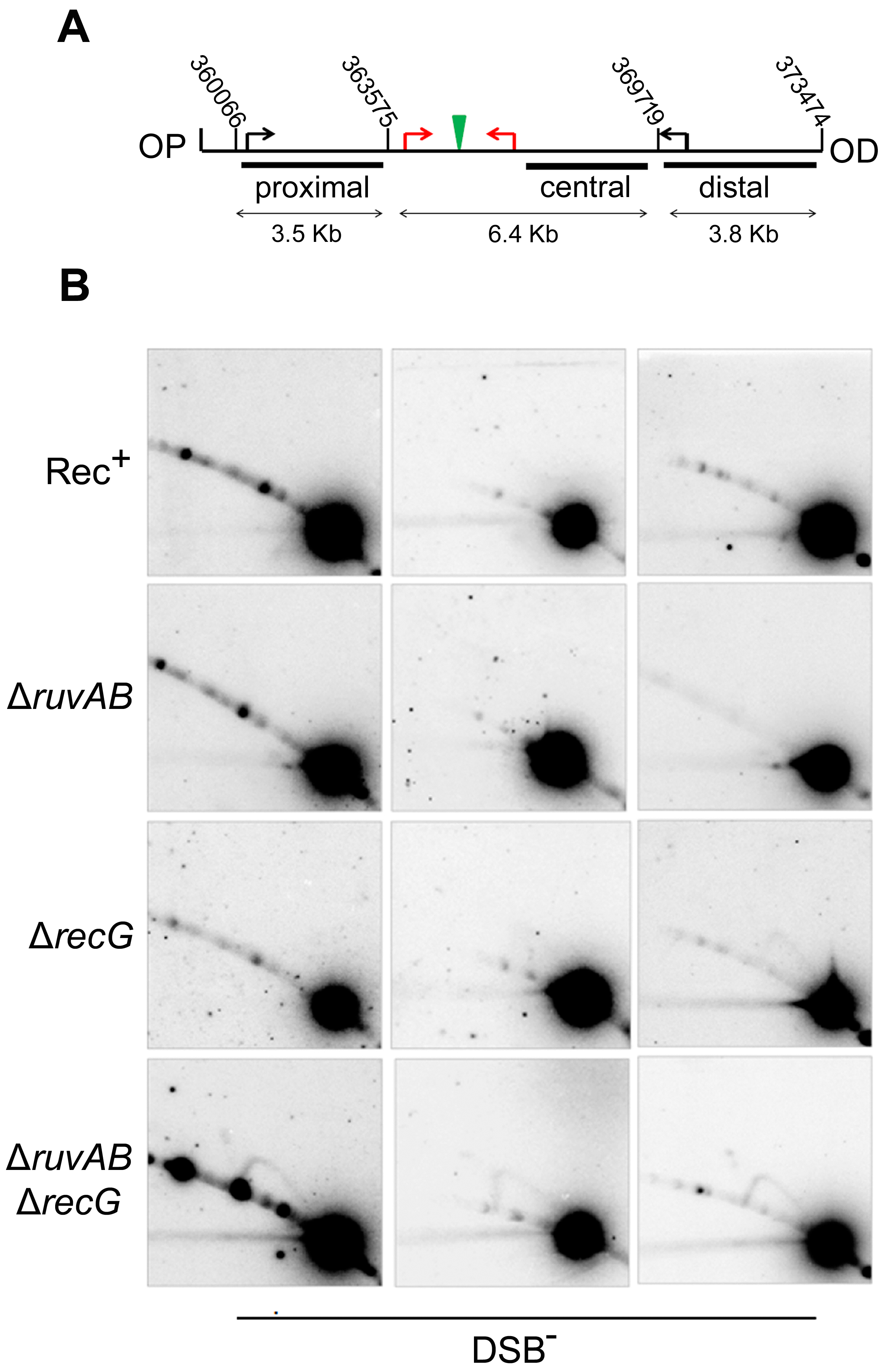

Supplement: Figure S3 — 2D agarose gel electrophoresis of DSB− condition. (A) Map of the chromosome showing the three SalI fragments around the DSB. The coordinates of the restriction sites are shown in black. The palindrome is shown as a green triangle and the 1.5 kb 3x χ arrays are shown as red arrows. Endogenous χ sites are shown as black arrows. The relative position of probes are represented by black rectangles. OP and OD indicate origin-proximal and origin-distal sides of the break, respectively. (B) Control 2D gels of strains not containing the palindrome, grown in the presence of 0.2% arabinose for 60 minutes. (DSB+ blots are shown in Figure 3C). Strains used; Rec+ (DL4201), ΔruvAB (DL4257), ΔrecG (DL4312), and ΔruvAB ΔrecG (DL4313). (TIF) [file pgen.1004485.s003.tif]
