# Supplementary material for: Branch Migration Prevents DNA Loss during Double-Strand Break Repair
Source: PLoS Genet. 2014 Aug 7;10(8):e1004485. doi: 10.1371/journal.pgen.1004485 (PMC4125073; doi:10.1371/journal.pgen.1004485)
Supplement: Table S1 — Table of E. coli strains. (DOCX) [file pgen.1004485.s004.docx]

**Table S1. Table of *E. coli* strains**

| **Strain** | **Genotype** | **Origin or Construction** |
| --- | --- | --- |
| MG1655 | F^-^ lambda^-^ *rph-1* | [32] |
| BW27784 | *lacI^q^* *rrnB3* *∆lacZ4787 ∆phoBR580 hsdR514* DE(*araBAD*)*567* DE(*rhaBAD*)*568* DE(*araFGH*) φ(∆P*_araE_* P*_CP18_-araE*) | [Khlebnikov et al., 2001] |
| N3793 | AB1157 Δ*recG263*::Kan^R^ | [25] |
| DL2006 | BW27784 ∆P*_sbcDC_* P*_BAD-sbcDC_*  *lacZ*::*pal246* *cynX*::Gm^R^ | [6] |
| DL2793 | DL2006 *tsx*::I-SceI_cs_ *proA*::I-SceI_cs_ | [6] |
| DL2075 | DL2006 *recA*::Cm^R^ | [6] |
| DL2155 | DL2006 Δ*recG263*::Kan^R^ | [6] |
| DL2801 | DL2006 Δ*ruvAB* | [6] |
| DL4464 | DL2801 Δ*recG263*::Kan^R^ | DL2801 X P1 N3793 Δ*recG263*::Kan^R^ |
| DL2573 | BW27784 *∆*P*_sbcDC_* P*_BAD_-sbcDC lacZ*^+^  *cynX*::Gm^R^ | [6] |
| DL2792 | DL2573 *tsx*::I-SceI_cs_ *proA*::I-SceI_cs_ | [6] |
| DL2605 | DL2573 *recA*::Cm^R^ | [6] |
| DL2610 | DL2573 Δ*recG263*::Kan^R^ | [6] |
| DL2800 | DL2573 Δ*ruvAB* | [6] |
| DL4465 | DL2800 Δ*recG263*::Kan^R^ | DL2800 X P1 N3793 Δ*recG263*::Kan^R^ |
| DL4184 | DL2793 *lacZ*::χ-array *mhpR*::χ-array | DL2793 PMGR sequentially with pDL4137 and pDL4138 |
| DL4243 | DL4184 Δ*ruvAB* | DL4184 PMGR with pDL2757 |
| DL4311 | DL4184 Δ*recG263*::Kan^R^ | DL4184 X P1 N3793 Δ*recG263*::Kan^R^ |
| DL4260 | DL4243 Δ*recG263*::Kan^R^ | DL4243 X P1 N3793 Δ*recG263*::Kan^R^ |
| DL4913 | DL4184 Δ*ruvC* | DL4184 PMGR with pDL2731 |
| DL4941 | DL4913 Δ*recG263*::Kan^R^ | DL4913 X P1 N3793 Δ*recG263*::Kan^R^ |
| DL4201 | DL2792 *lacZ*::χ-array *mhpR*::χ-array | DL2792 PMGR sequentially with pDL4137 and pDL4138 |
| DL4257 | DL4201 Δ*ruvAB* | DL4201 PMGR with pDL2757 |
| DL4312 | DL4201 Δ*recG263*::Kan^R^ | DL4201 X P1 N3793 Δ*recG263*::Kan^R^ |
| DL4313 | DL4257 Δ*recG263*::Kan^R^ | DL4257 X P1 N3793 Δ*recG263*::Kan^R^ |
| DL4914 | DL4201 Δ*ruvC* | DL4201 PMGR with pDL2731 |
| DL4942 | DL4914 Δ*recG263*::Kan^R^ | DL4914 X P1 N3793 Δ*recG263*::Kan^R^ |

References

Khlebnikov A, Datsenko KA, Skaug T, Wanner BL, Keasling JD (2001) Homogeneous expression of the P_BAD_ promoter in *Escherichia coli* by constitutive expression of the low-affinity high-capacity AraE transporter. Microbiology 147: 3241-3247.
