# Supplementary material for: Branch Migration Prevents DNA Loss during Double-Strand Break Repair
Source: PLoS Genet. 2014 Aug 7;10(8):e1004485. doi: 10.1371/journal.pgen.1004485 (PMC4125073; doi:10.1371/journal.pgen.1004485)
Supplement: Table S2 — Table of plasmids. (DOCX) [file pgen.1004485.s005.docx]

**Table S2. Table of Plasmids**

| **Plasmid** | **Description** | **Source** |
| --- | --- | --- |
| pTOF24 | Cm^R^ Kan^R^ *repA101*_(TS)_ Suc^s^ | [Merlin et al., 2002] |
| pDL2757 | pTOF24 + *∆ruvAB* knock-out fragment, Cm^R^  *repA101*_(TS)_, Suc^S^ | [6] |
| pDL2731 | pTOF24 + *∆ruvC* knock-out fragment, Cm^R^  *repA101*_(TS)_, Suc^S^ | Ewa Okely, unpublished |
| pDL4137 | pTOF24 + *lacZ*::χ-array knock-in fragment, Cm^R^  *repA101*_(TS)_ Suc^S^ | This work |
| pDL4138 | pTOF24 + *mhpR*::χ-array knock-in fragment, Cm^R^ *repA101*_(TS)_ Suc^S^ | This work |

References

Merlin C, McAteer S, Masters M (2002) Tools for characterization of *Escherichia coli* genes of unknown function. J Bacteriol 184: 4573-4581.
