# Supplementary material for: Branch Migration Prevents DNA Loss during Double-Strand Break Repair
Source: PLoS Genet. 2014 Aug 7;10(8):e1004485. doi: 10.1371/journal.pgen.1004485 (PMC4125073; doi:10.1371/journal.pgen.1004485)
Supplement: Table S3 — Table of primers. (DOCX) [file pgen.1004485.s006.docx]

**Table S3. Table of Primers**

Restriction sites are underlined in bold.

| **Name** | **Sequence (5' to 3')** | **Purpose** |
| --- | --- | --- |
| *lacZ*Chi 1 | AAA AA**C TGC AG**G TCG GCA AAG ACC AGA CC | Used for the construction of pDL4137. |
| *lacZ*Chi 2 | CAG CGC GTG TCC ACC AGC TCA GCA TCG ACC ACC AGC GTC ACG ACG CGC TGT ATC GCT G |  |
| *lacZ*Chi 3 | TCG ATG CTG AGC TGG TGG ACA CGC GCT GGC TGG TTG TTA GCG CCG TGG CCT GAT TC |  |
| *lacZ*Chi 4 | AAA AA**G TCG AC**C ACG CTG ATT GAA GCA GAA G |  |
| *mhpR*Chi 1 | AAA AA**C TGC AG**C TGG CAC CCA GTT GAT CG | Used for the construction of pDL4138. |
| *mhpR*Chi 2 | GCG CAG ACT CGC TGG TGG TCA CAT GGC GGC TGG TGG CAA CGG GTA GCA AAA CAG ATC |  |
| *mhpR*Chi 3 | CGC CAT GTG ACC ACC AGC GAG TCT GCG CCC ACC AGC TAG CGC CGG AAG ATG CTT TTC |  |
| *mhpR*Chi 4 | AAA AA**G TCG AC**A GTG GTA TGG CCG ACA GAT G |  |
| Proximal F | GGT GTG TGG GTT AGG TCT GG | Used to make the "proximal" probe |
| Proximal R | GTG CAC GGC AGA TAC ACT TG |  |
| Central F | ATC GTC GTA TCC CAC TAC CG | Used to make the "central" probe |
| Central R | TTT CCA TGC GAG GTT AAA GG |  |
| Distal F | AGG GAC GCA TAC AGG AAC TG | Used to make the "distal" probe |
| Distal R | TCC AGC GAA TAC TGA TGA CG |  |
| *yagV* F | AAA GCC CAT CGT TAC AGG TG | Used to make the "*yagV*" probe |
| *yagV* R | ATG ATA GCT GGC GGG ATA TG |  |
| *lacZ* F | CTG GCG TAA TAG CGA AGA GG | Used to make the "*lacZ*" probe |
| *lacZ* R | CAT GAC CTG ACC ATG CAG AG |  |
| *araJ* F | CAG CGT CAG CAT CAT ACC TC | Used to make the "*araJ*" probe |
| *araJ* R | GCC GAA TTT GGC ATT ATG G |  |
| *cysN* F | CGG TTG ATT GAC AAA TGC AC | Used to make the "*cysN*" probe |
| *cysN* R | ATC GCG TCA ATG TAC CCT TC |  |
| *codB* F | GGC CTG GAC ATT AAA AG | Used to make the "*codB*" probe |
| *codB* R | TGC TTC CAG TTC GGT TGT CC |  |
| *ykgK* F | TAC CAC GCC CCT AAT ATT GC | Used to make the "*ykgK*" probe |
| *ykgK* R | TTA CGT GTC GCA TCG CTA TC |  |
| pKO F | AGG GCA GGG TCG TTA AAT AGC | Amplifies across the PstI/SalI junction of pTOF24 |
| pKO R | AGG GAA GAA AG GAA AGG AG |  |
