# Supplementary material for: Branch Migration Prevents DNA Loss during Double-Strand Break Repair
Source: PLoS Genet. 2014 Aug 7;10(8):e1004485. doi: 10.1371/journal.pgen.1004485 (PMC4125073; doi:10.1371/journal.pgen.1004485)
Supplement: Protocol S1 — Construction of strains. (DOCX) [file pgen.1004485.s007.docx]

**Protocol S1. Construction of strains**

Strains containing the P*_BAD_-sbcDC* construct, which allows for the expression of the endonuclease, SbcCD using arabinose, are derivatives of BW27784, a strain that has been genetically altered for the homogeneous expression of the P*_BAD_* promoter [Khlebnikov et al., 2001]. Mutations in the *ruv* genes were made using plasmid-mediated gene replacement (PMGR) (plasmids are listed in Table S2), while mutations in *recG* were achieved by P1 transduction of the Δ*recG263*::Km^R^ mutation from N3797 [25; Link et al., 1997; Merlin et al., 2002]. Mutations were checked by PCR and mutant phenotypes were confirmed by loss of viability following exposure to UV-light.

References

Khlebnikov A, Datsenko KA, Skaug T, Wanner BL, Keasling JD (2001) Homogeneous expression of the P_BAD_ promoter in *Escherichia coli* by constitutive expression of the low-affinity high-capacity AraE transporter. Microbiology 147: 3241-3247.

Link AJ, Phillips D, Church GM (1997) Methods for generating precise deletions and insertions in the genome of wild-type *Escherichia coli*: application to open reading frame characterization. J Bacteriol 179: 6228-6237.

Merlin C, McAteer S, Masters M (2002) Tools for characterization of *Escherichia coli* genes of unknown function. J Bacteriol 184: 4573-4581.
