# Supplementary material for: Branch Migration Prevents DNA Loss during Double-Strand Break Repair
Source: PLoS Genet. 2014 Aug 7;10(8):e1004485. doi: 10.1371/journal.pgen.1004485 (PMC4125073; doi:10.1371/journal.pgen.1004485)
Supplement: Protocol S2 — Construction of pDL4137 and pDL4138. (DOCX) [file pgen.1004485.s008.docx]

**Protocol S2. Construction of pDL4137 and pDL4138**

The pTOF-*lacZ*::3x χ-array (pDL4137) plasmid was used to introduce a 44 bp cassette containing three repeats of the 8 base pair cross-over hotspot instigator sequence, Chi, into the *lacZ* gene, 1.5 Kb origin proximal of the palindrome. A 915 bp PCR fragment was amplified from *E. coli* MG1655 using primers *lacZChi* 1, 2,3, and 4. The resulting fragment contained the 44 bp Chi cassette in the centre, flanked by two regions of homology to the cassette's insertion locus. The PCR fragment was cut with PstI/SalI and ligated with PstI/SalI cut pTOF24 to replace the vector's Kan^R^ gene to generate the kanamycin sensitive pTOF-*lacZ*::3x χ-array (pDL4137) plasmid. The pTOF-*mhpR*::3x χ-array (pDL4138) plasmid was constructed as for pDL4137. Primers used were *mhpRChi* 1, 2,3, and 4. pDL4137 and pDL4138 were verified by sequencing using primers pKO F and pKO R, which are internal to the pTOF-backbone and amplify across the PstI/SalI junction.
